# Supplementary material for: Identification and functional analysis of non-coding regulatory small RNA FenSr3 in Bacillus amyloliquefaciens LPB-18
Source: PeerJ. 2023 May 15;11:e15236. doi: 10.7717/peerj.15236 (PMC10194069; doi:10.7717/peerj.15236)
Supplement: Supplemental Information 4 [file peerj-11-15236-s004.zip › KO/CK-vs-T1_map/map04016.html]

KEGG PATHWAY: MAPK signaling pathway - plant - Reference pathway


|  |  |
| --- | --- |
| **MAPK signaling pathway - plant - Reference pathway** |  |

[
Pathway menu
| Organism menu
| Pathway entry
| Show description
| User data mapping
]

Mitogen-activated protein kinase (MAPK) cascades, the evolutionarily conserved signaling modules in all eukaryotes, serve in transducing extracellular signals to the nucleus or cytoplasm for appropriate cellular responses, including cell division, differentiation, programmed cell death, and adaption to various stresses. In plant, several cascades are triggered by different biotic and abiotic stress stimuli such as pathogen infection, wounding, low temperature, drought, osmotic shock, high salinity, and reactive oxygen species. MAPK pathways also play pivotal roles in processes such as hormonal and developmental signaling. Multiple functional pathways initiated from distinct receptors often share the same kinase components while maintaining their signaling specificity. In contrast to those of animals and fungi, all the plant MAPK genes that have been reported belong to extracellular signal-regulated kinase (ERK) subfamily.

|  |  |  |
| --- | --- | --- |
| Reference pathway | 184% 150% 122% 100% 82% 67% 55% | 图片下载 |
